# Anatomical examination of the fibula: digital imaging study for osseointegrated implant installation

**DOI:** 10.1186/s40463-015-0055-9

**Published:** 2015-02-03

**Authors:** Yoshiaki Ide, Satoru Matsunaga, Jeffrey Harris, Daniel O’ Connell, Hadi Seikaly, Johan Wolfaardt

**Affiliations:** Department of Developmental and Regenerative Dentistry, School of Life Dentistry at Tokyo, The Nippon Dental University, 1-9-20 Fujimi, Chiyoda-ku, Tokyo, 102-8159 Japan; Department of Anatomy, Tokyo Dental College, 2-9-18 Misaki-cho, Chiyoda-ku, Tokyo, 101-0061 Japan; Division of Otolaryngology Head and Neck Surgery, Department of Surgery, Faculty of Medicine and Dentistry, University of Alberta, 1E4 WMC, 8440-112 Street, Edmonton, Alberta T6G 2B7 Canada; Institute for Reconstructive Sciences in Medicine (iRSM), Alberta Health Services/Covenant Health/University of Alberta, 1W-02, 16940-87 Avenue, Edmonton, Alberta T5R 4H5 Canada

**Keywords:** Fibula, Free vascularized fibular flaps, Dental implant, Jaw reconstruction, Osseointegrated implant, CT, CT scan analysis, Fibula anatomy

## Abstract

**Background:**

Free vascularized fibular flaps are commonly used in jaw reconstruction. CT scan images of the fibula are used in digital planning of jaw reconstruction. In order to fully describe the anatomy of the fibula, an imaging study of the fibula was undertaken. The purpose of the present study was to examine the anatomical structure of the fibula using patient CT images.

**Methods:**

The CT scan images of fibulae of 20 patients were used for the study. The results of the analysis showed that, of the widths, the anterior border of the fibula to the posterior surface was the largest dimension. The shape type analysis showed that the triangular type was most prominent near the head of the fibula, and the irregular type was most prominent towards the lateral malleolus.

**Results:**

The results of height and width related to the long axis of implant installation showed that the width of the central section was the largest. With respect to the length of available bone volume, the length near the lateral malleolus was larger than that near the head of the fibula. The results showed that there were significant differences in size between male and female fibulae.

**Conclusion:**

The present study provides a CT scan based analysis of the anatomy of the fibula. Important information for the optimal site of installation of osseointegrated implants in fibular free flap reconstructions is also provided.

## Background

Free vascularized fibular flaps (FVFF) have been widely adopted ever since Taylor et al. [1] used this method to reconstruct the tibia in 1975. FVFF is also used in maxillofacial regions, since reconstruction of the anatomic dental arch, oral functions, and facial aesthetics are better than with ilium and scapula grafts [2-5]. Osseointegrated implant use in FVFFs has also become common in recent years [6-8], resulting in improved outcomes of mastication, speech, and swallowing functions [9,10]. For jaw reconstruction, the fibula is harvested from the donor site by measuring upwards from the lateral malleolus. The point of sectioning the fibula is typically at 8–10 cm above the lateral malleolus [11].

Successful FVFF requires an understanding of fibular anatomy. As a result, a number of reports have been published on measurements of cadaver fibulae [12-15]. The use of osseointegrated implants in the fibula usually involves trimming a sharp anterior border (anterior margin) and then installing either a regular diameter (4.3 mm φ) or a narrow diameter implant (3.5 mm φ) along the axis from the anterior border of the fibula to the posterior aspect. Analysis of fibular shape with reference to these processes is extremely important from a clinical perspective. While there are reports that take anatomical considerations into account for installation of osseointegrated implants, the information is limited.

When performing osseointegrated implant installation, the dimension of the site for accomodation of the long axis diameter of the osseointegrated implant may be measured on preoperative CT images, so that appropriately sized implants can be chosen. Consequently, an understanding of the dimensions of the fibula as assessed from CT images is important for implant treatment. This becomes all more so when fully digitally derived 3D constructs with fully guided surgery are used in advanced jaw reconstruction procedures.

The objective of the present study was to investigate the anatomical characteristics of fibulae with a view toward implant treatment. Using preoperative CT data from patients undergoing lower jaw FVFF reconstruction, the anatomical characteristics (differences according to site and gender) at the site of implant installation, as well as the available bone volume for installing the implants, was investigated.

## Methods

### Patients

The research was performed with the approval of the University of Alberta Health Research Ethics Board (Date of Approval 1st Feb 2013, Project # Pro00036132). In the present study, 40 fibulae (20 in males, 20 in females) in 20 adult patients (10 males [mean: 48.0; range: 22–72], 10 females [mean: 64.2; range: 57–77]) were studied. The 20 patients underwent preoperative CT imaging at the University of Alberta Hospital before FVFF reconstruction.

### CT scanning

The patients were imaged using a SOMATOM Definition Flash medical CT system (Siemens, Oakville, Canada). Imaging was performed with a 120 kV tube voltage, 90 mA tube current, 1 mm slice thickness, and 0.66 mm pixel size.

### Image processing for measurements

First, DICOM data were imported into Mimics 13.1 (Materialise, Leuven, Belgium), and the fibula was modeled using the CT threshold values (Min, 226; Max, 3071) for the bone data (Mimics default CT values). The model was exported as STL data. Next, the 3D fibula image from the STL file was viewed on screen using Geomagic Qualify 2013 (Geomagic, Morrisville, NC, USA). Finally, cross-sections were created for observation, and the measurements were taken as described below at each cross-section.

### Measurements

#### Cross-section

**Figure 1:**
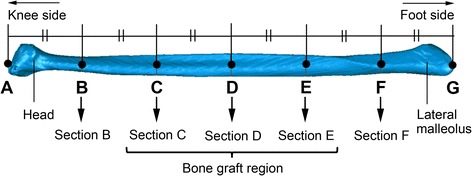
**Position of the analyzed transverse sections of the fibulae.**

The apex of the head of the fibula was point A, and that of the lateral malleolus was point G. The length between these two points was divided into six equal segments, and each of the dividing points from the apex of the head of the fibula was denoted B, C, D, E, and F (Figure 1). A cross-section perpendicular to the axis of the fibula was then created at each of points B, C, D, E, and F, and these sections were denoted as B, C, D, E, and F. Geomagic Qualify 2013 was used to define these sections and produce the measurements described below.

#### Length of fibula

The full length of the fibula was measured from the apex of the head of the fibula (point A) to the apex of the lateral malleolus (point G) (Figure 1). To confirm that sections C, D, and E were part of the bone graft region, their distances from the apex of the lateral malleolus were calculated from the full length of the fibula.

#### Width from the margins of the fibula to their opposing surfaces

**Figure 2:**
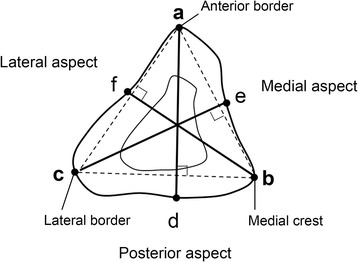
**Measuring the width from the margins of the fibula to their opposing surfaces.**

To obtain an overview of the entire fibula, length measurements of the fibula cross-sections at B, C, D, E, and F were performed, in accordance with the measurement system used by Matsuura [13]. As shown in Figure 2, the anterior border was defined as point a, the medial crest was defined as point b, and the lateral border was defined as point c. A perpendicular line was drawn from point a to line b–c, and the intersection of its extension with the posterior aspect was defined as point d. Similarly, a perpendicular line was drawn from point c to line a–b, and the intersection of its extension with the medial aspect was defined as point e. A perpendicular line was drawn from point b to line a–c, and the intersection of its extension with the lateral aspect was defined as point f. The distances a–d, c–e, and b–f were measured.

#### Cross-section shapes

**Figure 3:**
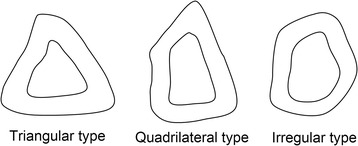
**Classifying transverse shape type.**

Drawing on the method of Matsuura et al. [13], the shapes of the cross-sections at C, D, and E of the bone graft region were categorized into three types: triangular, quadrilateral, and irregular (Figure 3).

#### Height and width related to the long axis of installation of the implant

**Figure 4:**
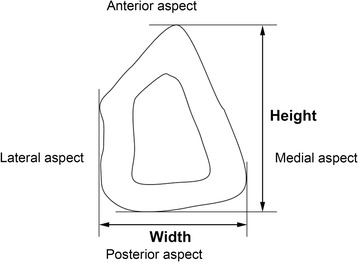
**Measuring height and width related to the long axis of implant installation.**

Drawing on the method of Frodel et al. [14], the height and width at sections C, D, and E were measured with reference to the long axis of installation of the implant (Figure 4).

#### Length of available bone volume for the osseointegrated implant installation

**Figure 5:**
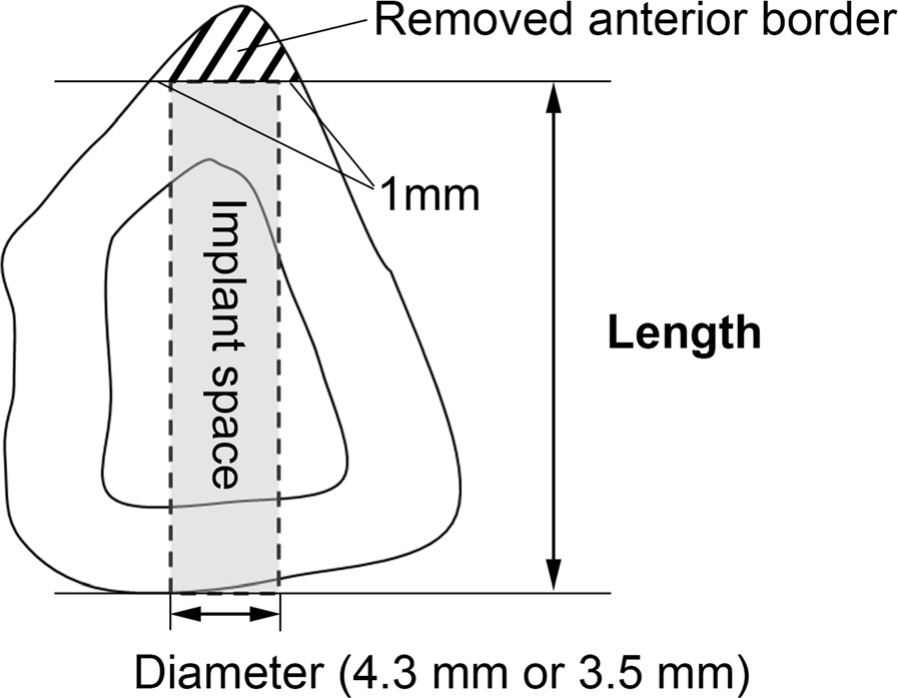
**Measuring length of available bone volume for the osseointegrated implant installation.**

The length available for implantation with the standard (4.3 mm φ) and narrow (3.5 mm φ) implants at sections C, D, and E was measured. As shown in Figure 5, dimension to account for 1 mm bone margin on both sides of the implant (lateral and medial aspects) was defined, and the anterior border was adjusted to the defined bone level.

### Statistical analysis

Statistical analysis was performed using SPSS (IBM, Armonk, NY, USA). For all data, the Bonferroni test was used to compare the differences between each section or each measured site, and Student’s *t*-test was used to examine sex differences.

## Results

### Length of the fibula

Table 1 shows the results of the measurements of fibula length from the apex of the head of the fibula (point A) to the apex of the lateral malleolus (point G), as well as the calculated lengths from the apex of the lateral malleolus to each of the sections B, C, D, E, and F. The mean fibula length, which was 387.4 ± 23.7 mm in male patients and 361.5 ± 12.3 mm in female patients, was significantly longer in the male patients than in the female patients.

**Table 1:** **Length of Fibula (mm)**

**Gender**	Full length*	**Position (Length from the apex of lateral malleolus)**				
		**Section B**	**Section C**	**Section D**	**Section E**	**Section F**
Male	387.4 ± 23.7	322.3 ± 19.7	258.3 ± 15.8	193.7 ± 11.8	129.1 ± 7.9	64.6 ± 3.9
Female	361.5 ± 12.3	301.3 ± 10.2	241.0 ± 8.2	180.8 ± 6.1	120.5 ± 4.1	60.3 ± 2.0

*The mean fibula length was significantly longer in the male patients than in the female patients (P < 0.01).

Investigations of the position of each section showed that the lengths from the apex of the lateral malleolus to sections F, E, D, C, and B were 64.6 ± 3.9 mm, 129.1 ± 7.9 mm, 193.7 ± 11.8 mm, 258.3 ± 15.8 mm, and 322.3 ± 19.7 mm, respectively, in the male patients and 60.3 ± 2.0 mm, 120.5 ± 4.1 mm, 180.8 ± 6.1 mm, 241.0 ± 8.2 mm, and 301.0 ± 10.2 mm, respectively, in the female patients. FVFF is performed using an area approximately 20cm in length, beginning approximately 9 cm from the malleolus. Therefore, the results confirmed that sections C, D, and E in both the male and female patients were part of the potential bone harvest region.

### Width from margins of the fibula to their opposing surfaces

**Figure 6:**
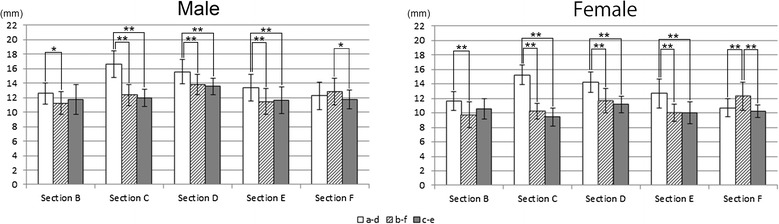
**Comparing a–d, c–e, and b–f in width from the margins of the fibula to their opposing surfaces.** ** P < 0.01, * P< 0.05.

Figure 6 shows the results of a comparison of the widths from the margins (anterior border, medial crest, lateral border) to their opposing surface (posterior surface, medial surface, lateral surface) at sections B, C, D, E, and F. At sections C, D, and E, a–d in both the male and female patients was significantly longer than b–f and c–e. At section B, a–d in both the male and female patients was significantly longer than b–f, but it was not significantly different from c–e. At section E, b–f tended to be the longest in both the male and female patients. These results showed that a–d at sections C, D, and E (the bone graft region) was the longest compared with the other widths.

A comparison of sections B, C, D, E, and F between male and female patients showed that the values for the male patients were significantly greater at all sites (P < 0.01).

### Cross-section shapes

**Figure 7:**
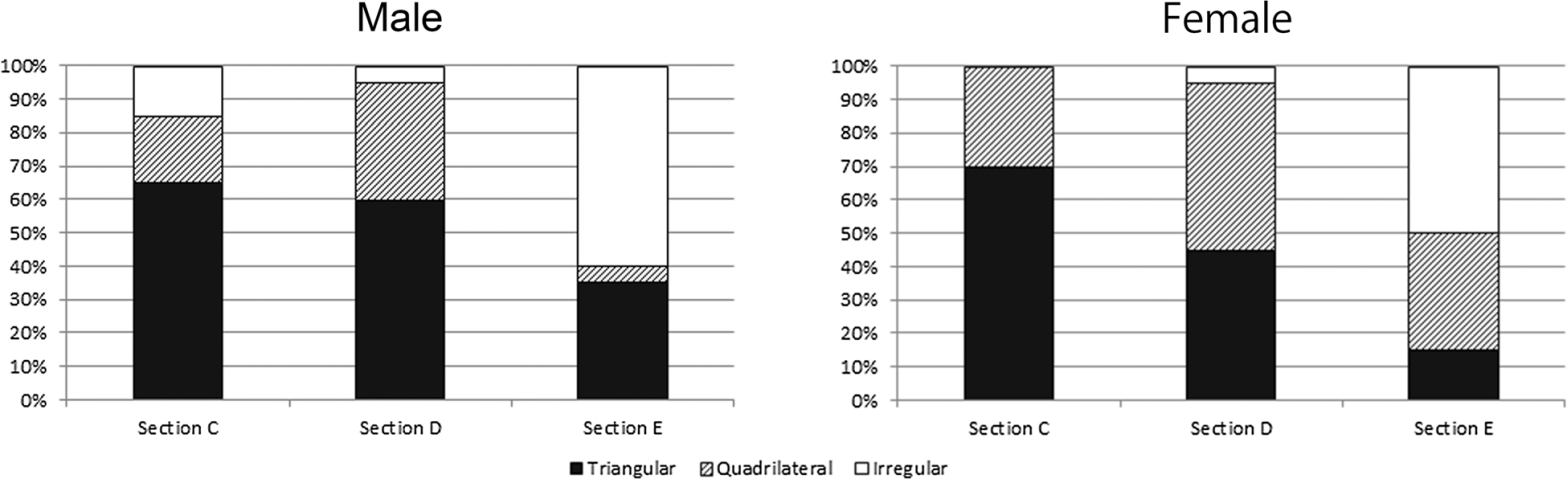
**Evaluation of the cross-section shapes of the fibula.**

Figure 7 shows the evaluation by shape at sections C, D, and E. In the male patients, the shapes were triangular in 65%, quadrilateral in 20%, and irregular in 15% of the patients at section C; triangular in 60%, quadrilateral in 35%, and irregular in 5% of the patients at section D; and triangular in 35%, quadrilateral in 5%, and irregular in 60% of the patients at section E. In the female patients, the shapes were triangular in 70%, quadrilateral in 30%, and irregular in 0% of the patients at section C; triangular in 45%, quadrilateral in 50%, and irregular in 5% of the patients at section D; and triangular in 15%, quadrilateral in 35%, and irregular in 50% of the patients at section E. These results showed that section C (towards the head of the fibula) was often triangular in both the male and female patients. Section D tended to be both triangular and quadrilateral in both the male and female patients. Section E (towards the lateral malleolus) often tended to be irregular.

### Height and width related to the long axis of implant installation

**Figure 8:**
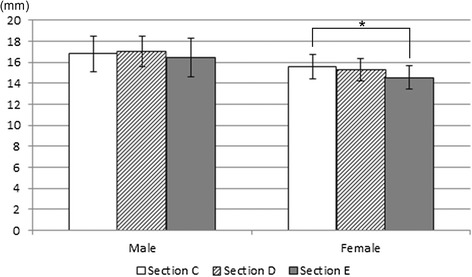
**Comparing height of sections C, D, and E related to long axis of implant installation.** ** P < 0.01, * P< 0.05.

**Figure 9:**
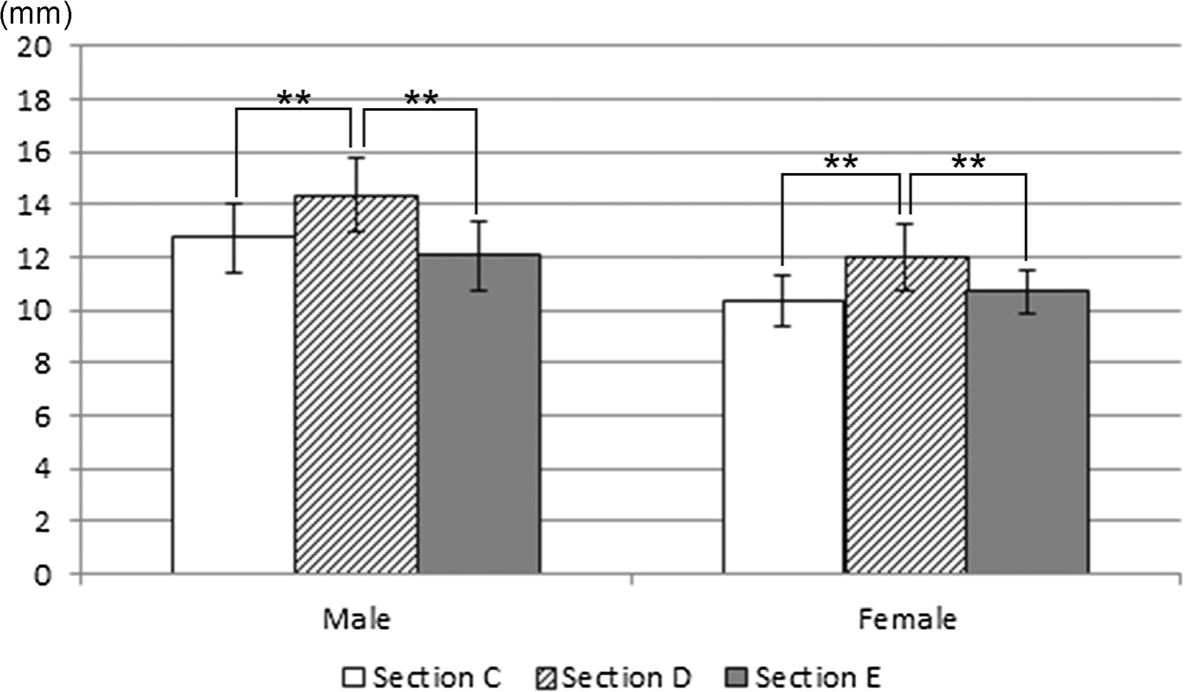
**Comparing width of sections C, D, and E related to long axis of implant installation.** ** P < 0.01, * P< 0.05.

Measurements were made of the fibula height and width as they relate to the long axis of implant installation at sections C, D, and E. A comparison of the heights at each section showed no significant differences in the male patients, and the value was significantly greater at section C than at section E in the female patients (Figure 8). A comparison of the widths at each section showed that values were significantly greater at section D than at sections C and E in both the male and female patients (Figure 9). A comparison of both height and width at each section between male and female patients showed that the male dimensions were significantly larger than the female dimensions at all sites (P < 0.01).

### Length of available bone volume for the osseointegrated implant installation

Table 2 shows the measurements of the length of available bone volume assuming the installation of regular (4.3 mm φ) and narrow (3.5 mm φ) implants. For the regular implants, the lengths at sections C, D, and E were 12.9 ± 2.2 mm, 13.4 ± 1.7 mm, and 14.5 ± 1.5 mm, respectively, in the male patients and 10.8 ± 2.0 mm, 10.9 ± 1.1 mm, and 12.0 ± 1.1 mm, respectively, in the female patients. For the narrow implants, the lengths at sections C, D, and E were 14.1 ± 2.1 mm, 14.3 ± 1.6 mm, and 14.8 ± 1.5 mm, respectively, in the male patients and 12.0 ± 2.0 mm, 11.9 ± 1.1 mm, and 12.5 ± 1.1 mm, respectively, in the female patients.

**Table 2:** **Length of available bone volume for the osseointegrated implant installation (mm)**

**Diameter of implant**	**Gender**	**Length**		
		**Section C**	**Section D**	**Section E**
4.3 mm (Regular type)	Male	12.9 ± 2.2	13.4 ± 1.7	14.5 ± 1.5
	Female	10.8 ± 2.0	10.9 ± 1.1	12.0 ± 1.1
3.5 mm (Narrow type)	Male	14.1 ± 2.1	14.3 ± 1.6	14.8 ± 1.5
	Female	12.0 ± 2.0	11.9 ± 1.1	12.5 ± 1.1

**Figure 10:**
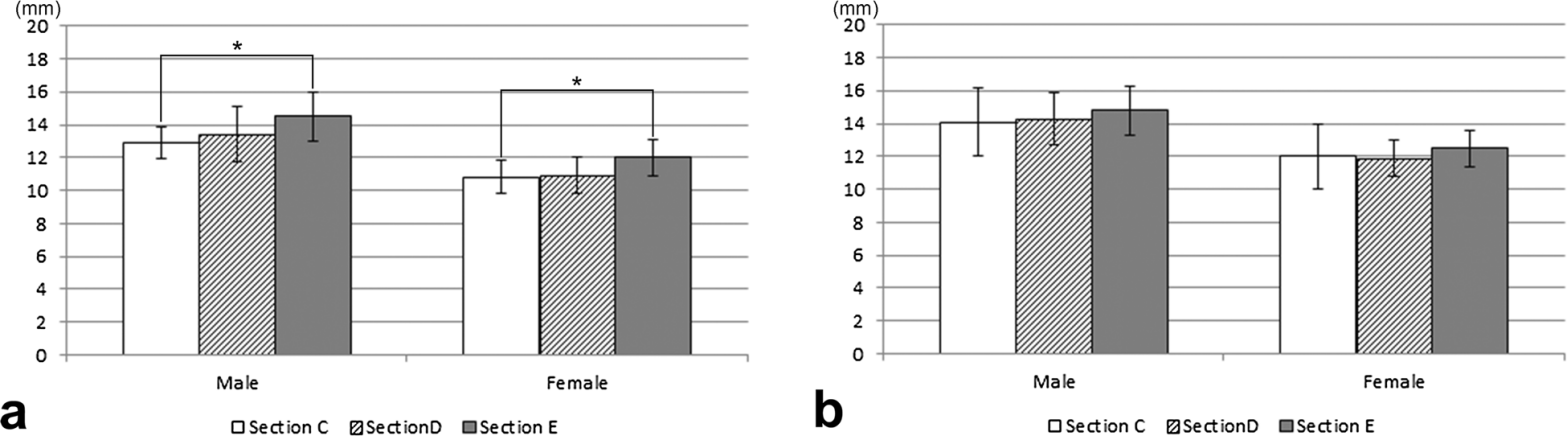
**Comparing length of available bone volume in sections C, D, and E for the 4.3 mm diameter (a) and 3.5 mm diameter (b) implant installation.** ** P < 0.01, * P< 0.05.

A comparison of these lengths at each section showed that with the regular diameter implant (4.3 mm φ), the length of available bone volume at section E was significantly greater than that at section C in both the male and female patients (Figure 10a). However, with the narrow diameter implant (3.5 mm φ), there was no significant difference between the sections in the length of the implant space (Figure 10b).

A comparison of the length of available bone volume for the regular (4.3 mm φ) and narrow (3.5 mm φ) implants between male and female patients showed that the lengths were significantly greater in male patients than in female patients at all sections (P < 0.01).

## Discussion

FVFF with installation of osseointegrated implants is now routinely performed for jaw reconstruction, and studies have shown high success rates and good functional recovery [6-10]. To achieve improved outcomes with treatments involving fibula implants, surgeons must possess an understanding of the shape characteristics of the fibula before the surgery.

The present study involved shape measurements based on data from medical CT imaging of patient fibulae. The advantages of measurements based on CT data are that cross-sections of interest can be easily observed in a non-destructive fashion, and a range of measurements are possible. Four parameters for investigation were established in cross-sections of the fibulae. In examining these measures through an imaging-based process, it is not known how dimensionally accurate the system is in relation to physical measurement on cadavers. The dimensional fidelity of the system is the subject of a study that is underway. Concerns with regard to the imaging study relate to issues such as the particular pixel size in images taken by medical CT, partial volume effects, or other factors. However, it was considered that the measured values can be compared since the values measured were acquired under the same condition. Statistical analyses were performed on differences at different sites, between patients of different sexes and to investigate the fibular shape characteristics. Preoperative planning for implant installation involves the measurement of the available bone volume on CT images and the selection of an appropriately sized implant. Hence, values for the length of available bone volume for osseointegrated implant installation were assessed.

### Length of fibula

FVFF is performed using an area approximately 20 cm in length running from a point approximately 9 cm from the lateral malleolus [11]. When the positions of sections B, C, D, E, and F from the lateral malleolus were calculated, section E was positioned at 129.1 ± 7.9 mm in the male patients and 120.5 ± 4.1 mm in the female patients, while section C was positioned at 258.3 ± 15.8 mm in the male patients and 241.0 ± 8.2 mm in the female patients. Therefore, the positions of sections C, D, and E were defined as part of the bone flap region and sections C, D, and E were investigated in detail.

### Width from the margins of the fibula to their opposing surfaces

The length of the installed implant is an important factor for implant stability [16]. Therefore, surgeons need to select on area with adequate bone volume for an implant length when installing an implant into the fibula. Osseointegrated implant installation in the fibula is usually performed along the axis from the anterior border towards the posterior surface, using the anterior border as an approximation of the alveolar crest. There are three margins in the fibula (anterior border, medial crest, lateral border) that could be used as the alveolar crest depending on the orientation of the fibula in relation to the vascular pedicle. Of particular interest is whether the possibility of using a long implant is greater when installing an implant along the axis from the anterior border than when doing so from the other margins. A comparison was made of the widths from these three margins to their opposing surfaces at sections B, C, D, E, and F. The measurements showed that, at sections C, D, and E, a–d was significantly longer than the other widths. This trend was not as marked at section B as it was at sections C, D, and E. At section F, b–f was significantly longer than the other widths. These results suggest that the region containing sections C, D, and E, which is used during bone flap reconstructions, provides a suitable axis of installation when considering implant stability, due to the distance from the anterior border to the posterior surface being greater than elsewhere.

### Cross-section shape

Anatomy textbooks [17] describe the fibular body as a triangular column and state that the cross-section of the fibula has 4 apices: the anterior border, the lateral border, the interosseous border, and the medial crest. To conduct more realistic evaluations in this study, the evaluation methods of Matsuura et al. [13] were used, and sections C, D, and E were categorized into three types: triangular, quadrilateral, and irregular. An understanding of shape trends at each site is valuable when establishing a treatment plan for fibula bone transplantation to the lower jaw and implant installation into the fibula. In the present study, section C was often triangular, and section E tended to be irregular. In other words, the study showed that the section closer to the head of the fibula is commonly triangular, and that towards the lateral malleolus tends to be irregular. Furthermore, compared with the quadrilateral and irregular shapes, the triangular shape has a more acute angle at the anterior border. Triangular shapes are better subjected to an elective ostectomy of the bony crest when installing implants. The reason for trimming the bony crest is to create a platform of bone of adequate width to accommodate the implant.

### Height and width related to implant long axis of installation

The fibula height and width at sections C, D, and E were measured with reference to the implant long axis of installation. In terms of height, a difference between sections C and E was observed in the female patients (P < 0.05), but no other significant differences were seen among sections. In terms of width, however, the fibulae at section D were significantly wider than those at the other sections (P < 0.01). Accordingly, there were no marked differences in height at the site of the fibula implant to be inserted, and the central portion of the fibula tended to be wider. This may be useful information when selecting the donor site on the fibula.

### Length of available bone volume for osseointegrated implant installation

When installing implants into the fibula, surgeons need to consider the characteristics of the available bone volume for installation. To perform more realistic measurements, the installation of regular (4.3 mm φ) or narrow (3.5 mm φ) osseointegrated implants was assumed. When installing implants into the fibula, preparing the site involves trimming the fibula alveolar crest to secure adequate width to install an osseointegrated implant. In the present study, the trimmed portion from the area measured was excluded, leaving a margin of 1 mm on both sides (lateral and medial aspects) of the implant.

The measurements from the present study were made using patient CT images, raising the possibility of measurement errors from partial volume effects or the particular pixel size used in the diagnostic CT imaging. The measurement error is likely small since the anterior border section, where errors are prone to occur, was excluded from the area measured. In addition, as CT imaging is normally used to select the appropriate implant for installation when planning implant treatment, it was considered that the values shown in this study are clinically meaningful. A comparison of the values at each section, assuming installation of a regular implant (4.3 mm φ), showed that, for both the male and female patients, were greater at section E than at section C. This suggests that sites closer to the lateral malleolus allow the installation of longer implants than do those towards the fibula head. When comparing the dimension of the bone to the implant long axis of installation, no bone height differences were seen at the various sites in the male patients, whereas in the female patients, the heights were significantly greater at sites near the head of the fibula than at those near the lateral malleolus. These results are attributed to differences in shape; at sites near the head of the fibula, the anterior border tends to be triangular with an acute angle rather than quadrilateral or irregular, so that sites near the head of the fibula involve more trimming of the bony crest prior to implant installation.

### Gender differences

All the measurements in the present study showed larger dimensions in the male patients than in the female patients. Therefore, this difference in available bone volume between the genders needs to be taken into consideration when installing an osseointegrated implant.

## Conclusion

In the present study, the fibula was investigated anatomically using patient CT images. The present study provides valuable information for the optimal site of installation of osseointegrated implants in FVFF reconstructions.

## Abbreviations

FVFF: Free vascularized fibular flaps
